# Essential role of the N-terminal region of TFII-I in viability and behavior

**DOI:** 10.1186/1471-2350-11-61

**Published:** 2010-04-19

**Authors:** Jaume Lucena, Susana Pezzi, Ester Aso, Maria C Valero, Candelas Carreiro, Pierre Dubus, Adriana Sampaio, Maria Segura, Isabel Barthelemy, Marc Y Zindel, Nuno Sousa, José L Barbero, Rafael Maldonado, Luis A Pérez-Jurado, Victoria Campuzano

**Affiliations:** 1 Genetics Unit, de Ciències Experimentals i de la Salut, Universitat Pompeu Fabra, Barcelona, Spain; 2 Laboratory of Neuropharmacology, Departament de Ciències Experimentals i de la Salut, Universitat Pompeu Fabra, Barcelona, Spain; 3 Beckman Institute, University of Illinois at Urbana Champaign, Urbana, USA; 4 Centro Nacional de Investigaciones Cardiovasculares Carlos III, Madrid, Spain; 5 Histologie et Pathologie Moleculaire, University of Bordeaux 2, Bordeaux, France; 6 Life and Health Sciences Research Institute, University of Minho, Braga, Portugal; 7 Centro de Investigación Biomédica en Red en Enfermedades Raras (CIBERER), Spain; 8 Department of Cell Biology and Development, Centro de Investigaciones Biológicas, Madrid, Spain; 9 Program in Molecular Medicine and Genetics, Hospital Universitari Vall d'Hebron, Barcelona, Spain

## Abstract

**Background:**

*GTF2I *codes for a general intrinsic transcription factor and calcium channel regulator TFII-I, with high and ubiquitous expression, and a strong candidate for involvement in the morphological and neuro-developmental anomalies of the Williams-Beuren syndrome (WBS). WBS is a genetic disorder due to a recurring deletion of about 1,55-1,83 Mb containing 25-28 genes in chromosome band 7q11.23 including *GTF2I*. Completed homozygous loss of either the *Gtf2i *or *Gtf2ird1 *function in mice provided additional evidence for the involvement of both genes in the craniofacial and cognitive phenotype. Unfortunately nothing is now about the behavioral characterization of heterozygous mice.

**Methods:**

By gene targeting we have generated a mutant mice with a deletion of the first 140 amino-acids of TFII-I. mRNA and protein expression analysis were used to document the effect of the study deletion. We performed behavioral characterization of heterozygous mutant mice to document *in vivo *implications of TFII-I in the cognitive profile of WBS patients.

**Results:**

Homozygous and heterozygous mutant mice exhibit craniofacial alterations, most clearly represented in homozygous condition. Behavioral test demonstrate that heterozygous mutant mice exhibit some neurobehavioral alterations and hyperacusis or odynacusis that could be associated with specific features of WBS phenotype. Homozygous mutant mice present highly compromised embryonic viability and fertility. Regarding cellular model, we documented a retarded growth in heterozygous MEFs respect to homozygous or wild-type MEFs.

**Conclusion:**

Our data confirm that, although additive effects of haploinsufficiency at several genes may contribute to the full craniofacial or neurocognitive features of WBS, correct expression of *GTF2I *is one of the main players. In addition, these findings show that the deletion of the fist 140 amino-acids of TFII-I altered it correct function leading to a clear phenotype, at both levels, at the cellular model and at the *in vivo *model.

## Background

Williams-Beuren syndrome (WBS) is among the most compelling genetic disorders of human development and behavior and is due to a recurring deletion of about 1,55-1,83 Mb containing 25-28 genes in chromosome band 7q11.23 [1,2]. Individuals with WBS manifest characteristic craniofacial dysmorphic features, cardiovascular problems, mild to moderate mental retardation and mild growth retardation [3]. The behavioral and cognitive profile of WBS individuals is characterized by relatively proficient language and face-processing skills, but serious impairments in visuo-spatial and numerical abilities. Personality traits include overfriendliness and charismatic speech. Hyperacusis or algiacusis is an almost constant feature. The roots of the mental and cognitive aspects of WBS probably lie in disruption of normal neurodevelopment, because brain morphology and neural organization are abnormal [4]. A firm pathogenic mechanism has been established for the cardiovascular problems that are known to be caused by haploinsufficiency for the elastin gene [5]. Additional candidates for involvement in several aspects of the WBS phenotype have been proposed on the basis of clinical-molecular correlations in a few patients with partial phenotypes and smaller deletions. Two members of the *GTF2I *gene family, *GTF2I *and *GTF2IRD1*, highly expressed during development and in normal neuronal tissues, are strong candidates for the craniofacial and some neurobehavioral features of WBS [6-9]. Homozygous loss of either the *Gtf2i *or *Gtf2ird1 *function in mice provided additional evidence for the involvement of both genes in the craniofacial and cognitive phenotype. Mice with a heterozygous deletion of exons 2-4 of *Gtf2ird1 *are less aggressive, more sociable and possibly less anxious [10-12]. However, any phenotype identified in a model of total deletion can potentially be assigned to the distal or the proximal half of the deletion indicating that loss of genes in both half-deletions may contribute to the phenotype [13]. Mouse models that resemble the WBS genetically and phenotypically are crucial to understand the roles haploinsufficient genes play in development, cognition and behavior. A great deal of information about possible functions of TFII-I has been obtained recently [9,12,14]. However, despite all these advances, the specific contribution of these genes to the phenotype and the underlying pathogenic mechanisms of the disease are not yet fully understood. Here, we have studied the *in vivo *effects of an in frame deletion of exon 2 of *Gtf2i*. The phenotypic characterization of this mouse model confirms that any alteration in the correct function of TFII-I contribute to important features displayed by WBS patients such as the craniofacial abnormalities and some neurobehavioral features including hyperacusis.

## Methods

### Generation of *Gtf2i*^*Δex*2 ^mutant mice

**Figure 1:**
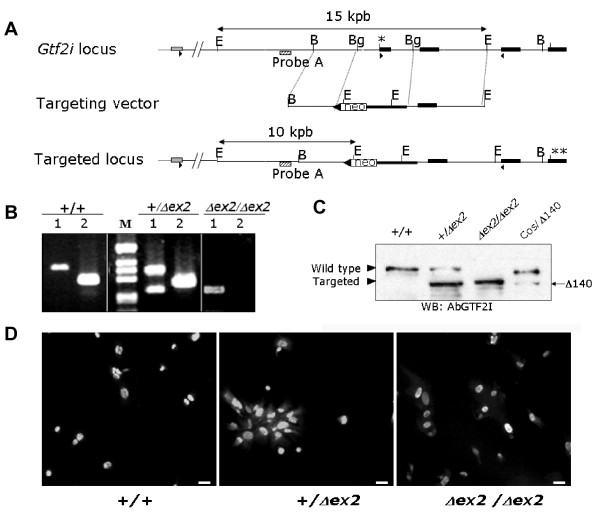
**Structure and expression of the *Gtf2i*^*Δex*2 ^mutant allele**. **A**. *Schematic genomic diagram of the targeting strategy*. **a**. Partial restriction map of wild-type genomic DNA encompassing exons 1 (grey box), 2, 3, 4 and 5 of the *Gtf2i *locus (filled boxes) (B, *BamHI*; E, *EcoRI; *Bg, *BglII*); the translation initiator sites in the wild type (*) and targeted (**) alleles are indicated. The arrows indicate the location of the primers used for RT-PCR analysis. The targeting vector contains the *PGK-neo *(open box, neo) and *lox511 *sequences (filled triangle). Predicted targeting allele after homologous recombination. The stippled box indicates the location of the probe used for Southern blot analysis, recognizing *EcoRI *DNA fragments of 14,7kbp (wild-type allele) and 9,7kbp (targeted allele). **B**. *RT-PCR analysis*. Using primers located in exons 1 and 4 (1) or 2 and 4 (2) of *Gtf2i *on mRNA from *Gtf2i*^*+/+*^, *Gtf2i*^*+/Δex*2 ^and *Gtf2i*^*Δex*2/*Δex*2 ^mice. Transcripts lacking exon 2 are observed in heterozygous and homozygous mutant mice (1), while no amplicon was obtained with exon 2 and 4 primers in *Gtf2i*^*Δex*2/*Δex*2 ^mice (2). **C**. ***Western blot analysis***. Total protein extracts from MEFs (*Gtf2i*^*+/+ *^; *Gtf2i*^*+/Δex*2 ^and *Gtf2i*^*Δex*2/*Δex*2^) and COS7 cells transfected with a *Gtf2i*^*Δex*2 ^cDNA lacking the first four exons were used for western blot analysis. We can observed that the truncated form present in *Gtf2i*^*+/Δex*2 ^and *Gtf2i*^*Δex*2/*Δex*2 ^extracts co-migrate with the artificial truncated form expressed in COS7 cells. **D**. ***Subcellular localization of endogenous TFII-I in MEFs***. The genotype of the cells is shown in the bottom. Equal nuclear localization of Δ140TFII-I and wild-type proteins was confirmed by DAPI staining in all cases. Scale bar: 10 μm.

We have targeted *Gtf2i *in ES cells by a strategy consisting in replacing exon 2 by a PGK-*neo *cassette (Figure 1A). Detailed information regarding the generation of the targeting vector as well as the PCR strategy to genotype mice can be found in Additional file 1: Methods. Animals were maintained under standard animal housing conditions in a 12-h dark-light cycle with free access to food and water. Animal care was in accordance with ethical guidelines (European Communities Council Directive 86/609/EEC) and approved by the Local Ethical Committee (IMAS-IMIM/UPF).

### Cell Culture and Immunocytofluorescence

We obtained and characterized MEFs following the previously described protocols [15]. All the experiments were made by triplicate. Spontaneous immortalization was carried out following a classical 3T3 protocol [16].

To detect endogenous and over-expressed TFII-I, immunocytofluorescence was performed in primary MEFs and transfected COS7 cells. After fixation and permeabilization, cells were stained with the specific designed anti-TFII-I rabbit polyclonal antibodies. Nuclear DNA was additionally stained with Hoechst 33258 (Molecular Probes). Microphotographs were taken using an Olympus BXS1 microscope with an epifluorescence and phase-contrast optics equipped with the Olympus DP71 camera and using the software Cell^B ^Digital Imaging system. A more detailed description can be found in Additional file 1.

### mRNA expression analysis

Total RNA from cells and tissue samples of mice was isolated using the TRIZOL reagent (Invitrogen) or RNeasy columns (Qiagen). Detailed protocols for RT-PCR analysis and mRNA quantification elsewhere as primers sequences are described in Additional file 1. Normalization of values has been made using housekeeping genes *Actin *and *Hsp70*. In all the cases, experiments were carried out in triplicate to ensure reliability.

### Protein analysis

Plasmids expressing mouse TFII-I wild type and Δ140TFII-I (β-and Δisoforms) were generated using a PCR-based strategy (TFIIWT and TFIIN140 as forward primers and a common reverse primer TFIIR) (see sequences in Additional file 1). PCR products were cloned into the pcDNA3.1 Directional TOPO Expression Kit (Invitrogen). Correct amplification of cloned products was tested by sequencing. Transient transfection of COS7 cells was carried out using Fugene reagent (Roche). Equal loading and transfer of samples were confirmed by staining the membranes with Ponceau S. After blocking with 3% BSA, membranes were incubated with the specific primary antibodies: anti-TFII-I; p21^Cip1^; p53 (Neo Markers) and α-tubulin (Ab-1; Oncogene Research). After incubation with the corresponding secondary antibodies, ECL-plus kit (Amersham Pharmacia) was used to visualize the protein.

### Histology and immunohistochemstry

E6.5-7.5 deciduas or adult tissues were surgically removed, fixed in 10% buffered formalin (Sigma), and embedded in paraffin using standard procedures. Sections (6 μm) were stained with hematoxylin and eosin (H&E). TUNEL assays were performed using the *in situ *cell death detection kit (Roche). Cell proliferation was determined by immunostaining with anti-Ki67 (Dako) according to the manufacturer's instructions. Brains were split into two hemispheres by a midsagittal section and processed for stereology, according to the procedure described previously [17]. More detailed description of stereology analysis can be found in Additional file 1.

### Behavioral characterization of mice

Mutants and wild-type littermate mice were tested in a battery of "primary screen" behavioral assays in the following order: wire hanging test, actimetry boxes, hot plate, lit-dark box, tail immersion, elevated plus maze and sound intolerance. The testing order was predetermined, with less stressful tests conducted before more stressful ones. The tests spam during 45 days with different time intervals between tests. Additional batches of mice were used for intruder test studies. Each batch of mice consisted of 15 male mice per genotype to ensure sufficient statistical power against variation of behavioral data. More detailed description of behavioral tests can be found in Additional file 1.

### Statistical analyses

Data were analyzed using a one-way ANOVA with genotype as between subjects' factor followed by *post hoc *comparisons (Tukey-Scheffé test) to detect statistical differences among levels. The alpha-corrected level for all tests was 0.05. All statistical tests were made under the R environment.

## Results

### *Gtf2i*^*Δex*2 ^mutant mice express a truncated TFII-I form

We have generated the *Gtf2i*^*Δex*2 ^mutant mice using gene targeting strategies. Exon 2 of *Gtf2i *was replaced by a PGK-*neo *cassette (Figure 1A). Almost 4% of the clones carried the correct mutation. Positive clones were aggregated with CD1 morulae to generate chimeras. Germ-line transmission was achieved by crossing chimeric males with CD1 females and correct mutation transmission was screened by Southern blot analysis.

RT-PCR analysis with primers located in the first and fourth exons revealed that removal of exon 2 generated an alternative shorter transcript (Figure 1B). Sequence of this transcript confirmed the absence of exon 2. Western blot analysis revealed that this transcript is translated in a shorter form of TFII-I (Figure 1C). Since this truncated form was recognized by the anti-TFII-I antibody, we hypothesized that the deletion of exon 2 containing the initiation codon abrogated to the use of a second in frame start codon. *In silico *analysis (Netstart) identified a putative in frame translation initiation site with suitable Kozak consensus sequence at exon 5, predicting a protein lacking the first 140aa (Δ140TFII-I). We generated an expression vector with a *Gtf2i *cDNA coding for such a truncated Δ140TFII-I (exons 5 to 35), and expressed it in COS7 cells. Figure 1C shows that the observed endogenous truncated form in the *Gtf2i*^*+/Δex*2 ^and *Gtf2i*^*Δex*2/*Δex*2 ^mice co-migrated with the artificial truncated protein, indicating that the ATG identified at exon 5 was indeed used as the start codon for translation in the targeted gene.

*In vitro *studies have shown that the deletion of the N-terminal 90 amino acids of a TFII-I isoform does not effect in the nuclear localization but affects its DNA binding ability [18]. By immunocytochemistry, we detected that both, endogenous and over-expressed mutant Δ140TFII-I was localized preferentially in the nucleus of primary MEFs and transfected COS7 cells (Figure 1D). Co-existence of wild-type and mutant forms does not affect their nuclear translocation. Both forms showed a diffuse patter of fluorescence without the formation of aggregates or any specific pattern distribution into the nucleus. Surprisingly, ChIP analysis demonstrates the ability of the truncated form to interact with endogenous DNA target sequences (see Additional file 2).

### Functional properties of the truncated TFII-I protein

We performed cotransfection followed by coimmunoprecipitation assays to evaluate if Δ140TFII-I form was able to dimerize with wild-type TFII-I and also to interact with known nuclear factors, as PARP1 [19]. As expected wild-type TFII-I was able to co-immunoprecipitate itself and PARP1, however, in the same conditions Δ140TFII-I form was unable to co-immunoprecipitate either wild-type TFII-I or PARP1 (see Additional file 2). These experiments suggest that at least for some targeted pathways, the truncated allele was working as a null allele.

Consequently, we attempted to test the transcriptional properties of the Δ140TFII-I compared with the wild type TFII-I protein by quantifying the endogenous and exogenous mRNA levels from several genes (*Cyclin D1*, c*-fos, Luciferase, Gapdh, Hsp70, β-actin *and *Gtf2i*) in arrested or serum stimulated MEFs and COS7 cells transfected with Δ140TFII-I-β or Δ140TFII-I-Δ constructs, using murine TFII-I-β and TFII-I-Δ and human TFII-I-Δ constructs as controls. Among the genes examined, c*-fos *and *Cyclin D1 *contain canonical Inr elements on the promoter region close to the transcription start sites and have been previously reported as substrates for TFII-I [20-22]. Expression of endogenous *Cyclin D1 *was increased when cells were transfected with TFII-I-Δ but not with the Δ140TFII-I-Δ form suggesting a lost of function for the truncated form at least for some of the defined target-genes. Concerning the TFII-I-β constructs we could not appreciate any significant expression changes between genotypes, indicating a lack of significant effect of TFII-I-β forms in the reported genes in the cellular models used (see Additional file 3).

### Growth properties of mutant mouse embryonic fibroblasts

**Figure 2:**
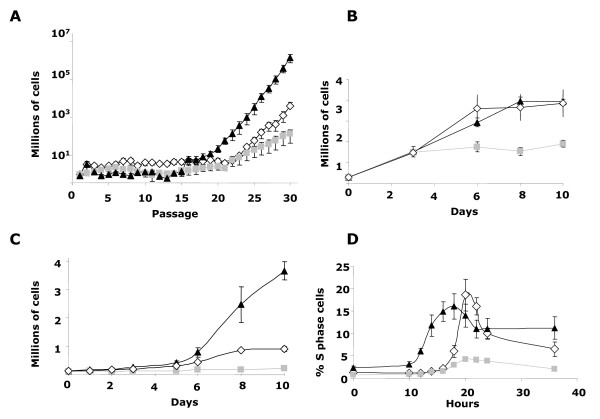
**Growth properties of *Gtf2i*^*Δex*2 ^mutant MEFs**. **A**. *Spontaneous immortalization*. Eleven independent MEF cultures showing early immortalization in *Gtf2i*^*Δex*2/*Δex*2 ^and later in *Gtf2i*^*+/Δex*2 ^cells. **B**. *Saturation rate*. 10^6 ^cells plated by triplicate and counted every three days until they reached the maximum score. A poorer saturation rate was observed in *Gtf2i*^*+/Δex*2 ^cells. **C**. *Proliferation of immortal MEFs*. Three independent experiments were performed with all cell types and each time point was done by triplicate. The fastest proliferation was observed in *Gtf2i*^*Δex*2/*Δex*2 ^while *Gtf2i*^*+/Δex*2 ^cells performed the worst. **D**. ***Re-entry into S-phase after serum deprivation***. The percentage of cells in S-phase was measured at the indicated times after serum stimulation. Again *Gtf2i*^*+/Δex*2 ^MEFs showed the lowest rate of S-phase re-entry. *Gtf2i*^*+/+*^, open rhombus; *Gtf2i*^*+/Δex*2 ^grey squares; *Gtf2i*^*Δex*2/*Δex*2 ^black triangles. All values are expressed as mean ± s.d.

Next, we decided to test whether Δ140TFII-I may modify the direct link between mitogen-dependent signaling and changes in nuclear gene expression that govern cellular proliferation and cell division. Eleven independent fibroblast cultures derived from six different embryos were grown following the classical 3T3 protocol to obtain fully immortalized clones [16]. As illustrated in Figure 2A, *Gtf2i*^*Δex*2/*Δex*2 ^MEF cultures immortalized around eight passages earlier than *Gtf2i*^*+/+ *^and *Gtf2i*^*+/Δex*2 ^cultures independently of the chosen immortalization pathway (p53 dependent or not).

In order to abrogate any effect of immortalization pathway in growth properties of cellular models, we performed the next experiments in cultures immortalizes following disruption of p53 pathway. *Gtf2i*^*+/Δex*2 ^MEF cultures did not growth at high density. Indeed, the number of *Gtf2i*^*+/Δex*2 ^cells in saturated cultures was 1.9 ± 1.5 × 10^6 ^per 10 cm dish (n = 3), whereas wild-type and *Gtf2i*^*Δex*2/*Δex*2 ^MEFs reached saturation levels that were two times higher (3.8 ± 6.5 and 4.1 ± 0.15 × 10^6 ^per 10 cm dish, n = 3, respectively) (Figure 2B). *Gtf2i*^*+/Δex*2 ^MEF cultures also proliferated less efficiently than controls' (Figure 2C) and had reduced plating efficiency opposite to the properties shown by *Gtf2i*^*Δex*2/*Δex*2 ^cultures. Whereas plating 4000 *Gtf2i*^*+/Δex*2 ^MEFs in a Petri dish yielded only 21 ± 3.5 colonies, the same amount of *Gtf2i*^*+/+ *^or *Gtf2i*^*Δex*2/*Δex*2 ^MEFs led to 114 ± 7.1 and 142 ± 4.2 colonies, respectively. Finally, quiescent *Gtf2i*^*+/Δex*2 ^MEFs re-entered the cell cycle with normal kinetics upon serum stimulation (Figure 2D). However, the proportion of cells that entered to the S phase was reduced by about 70% when compared with *Gtf2i*^*+/+ *^or *Gtf2i*^*Δex*2/*Δex*2 ^MEFs.

### Homozygous mutant mice have highly reduced viability and fertility, and abnormal craniofacial morphology

**Figure 3:**
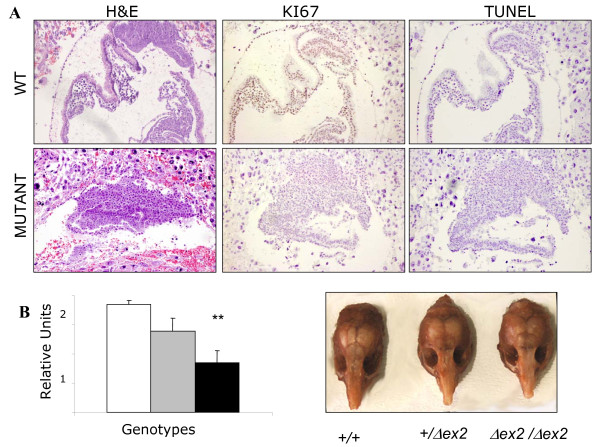
**Embryonic abnormalities and craniofacial dysmorphology in *Gtf2i*^*Δex*2 ^mutant mice**. **A**. *H&E staining, Ki67 labeling and TUNEL assay*. Sections from normal (top) and abnormal (bottom) E7.5 embryos derived from crosses between *Gtf2i*^*+/Δex*2 ^females and *Gtf2i*^*Δex*2/*Δex*2 ^males. Abnormal embryos lacking detectable embryonic layers by H&E staining are likely to correspond to nonviable *Gtf2i*^*Δex*2/*Δex*2 ^embryos. Normal embryos displayed high levels of Ki67-positive cells while they are no detectable in abnormal embryos. No differences were observed in the TUNEL staining. **B**. *Morphologic abnormalities in Gtf2i mice at 8 weeks of age*. (Left) The histogram shows the relative average distance from the tip of the nose to the proximal occipital suture (n = 10 per genotype). Pair wise comparisons revealed statistically significant differences between genotypes (Tukey test; *P *= 0.003 and *P *= 0.048 for *Gtf2i*^*Δex*2/*Δex*2 ^and *Gtf2i*^*+/Δex*2 ^relative to *Gtf2i*^*+/+*^, respectively). (Right) representative photograph of the dissected cranial structure per genotype is shown. A shorter snout could be clearly appreciated in the *Gtf2i*^*Δex*2/*Δex*2 ^mice. *Gtf2i*^*+/+*^, open; *Gtf2i*^*+/Δex*2^, grey; and *Gtf2i*^*Δex*2/*Δex*2^, black squares.

The total absence *Gtf2i *is lethal in embryonic development between E8.5 and E12.5 [12]. Abnormal Mendelian frequencies of the expected genotypes in different crosses were found at birth, with a clear reduction of *Gtf2i*^*Δex*2/*Δex*2 ^animals (Table 1). By contrast, surviving *Gtf2i*^*Δex*2/*Δex*2 ^mice appeared to be grossly normal, indistinguishable from their wild-type or heterozygous littermates. To investigate the role of *Gtf2i*^*Δex*2 ^mutation in mouse development, we first analyzed the genotype frequencies of embryos at 9.5-12.5 dpc. In contrasts to completed *Gtf2i *KO mice, at 9.5 dpc dissected *Gtf2i*^*Δex*2/*Δex*2 ^embryos were morphologically indistinguishable of heterozygous or wild-type littermates but were already underrepresented from the expected Mendelian ratio (Table 1), suggesting a much higher rate of early lethality. Histological examination of decidual swellings at E6.5-8 dpc revealed a significant percentage of highly disorganized embryos with gross morphological alterations [Normal/Abnormal = 58(72.5%)/22(27.5%)]. Only structures related to the ectoplacental cone and trophoblast giant cells could be morphologically identified (Figure 3A). Loss of the epiblast in defective embryos appeared to be a consequence of decreased cell proliferation, as determined by a marked reduction of Ki67 immunostaining. No increased apoptosis was revealed by TUNEL staining despite that a significant number of *Gtf2i*^*Δex*2/*Δex*2 ^animals were lost before E7.5 dpc (Figure 3A).

**Table 1:** Genotypes' distribution during embryonic development and at birth

Mating				Genotype		
			
Male	Female	Age	+/+	*+/Δex2*	*Δex2/Δex2*	*P*
*Δex2/Δex2*	*+/Δex2*	E8.5-E12.5	-	64	6	8.3 × 10^-13^
		At Birth	-	131	22	5.5 × 10^-20^
*+/Δex2*	*+/Δex2*	At Birth	167	340	48	3.7 × 10^-18^

On physical exam, gross anatomy of mutant mice appeared to be normal, with normal body size and weight. However, in a 70-87% CD1 background a more detailed evaluation revealed an obvious craniofacial phenotype in *Gtf2i*^*Δex*2/*Δex*2 ^mice, more subtle in *Gtf2i*^*+/Δex*2 ^animals, consisting in a shorter nose and wider nasal bridge (Figure 3B). Specific measurements of cranial morphology (distance from the occipital suture to the tip of the nasal bone) revealed significant differences among genotypes (*P *= 0.004). These features are quite similar to the craniofacial anomalies also present in the homozygous *Gtf2ird1 *KO mice [10]. Despite the craniofacial alteration, brain morphology appeared anatomically normal and brain weight is similar to wild-types or heterozygous littermates. Because the WBS phenotype suggests prominent functional alterations in the hippocampal function, we searched for putative developmental neuroanatomical defects by stereological analysis of volumes and total cell numbers. We analyzed global brain and three areas of the hippocampus (CA1, CA3 and the dentate gyrus) in five adult male animals per genotype. No significant abnormalities were observed in the mutant animals with respect to the controls (data not shown).

During the normal breeding of the animals we observed a reduced fertility period in both males and females homozygous mutant mice. In both genders no more than 4 consecutive litters were obtained with a progressive reduction in total pups (see Additional file 4). Homozygous mother behavior was completely normal and pup development is not affected. Anatomical examination of old mice reveal smaller and atrophy testis in the 80% of homozygous males analyzed but any anatomy alteration that could explain affected fertility was observed in ovaries or uterus of mutant females.

### Behavioral alterations in adult *Gtf2i*^*+/Δex2 *^mice

**Figure 4:**
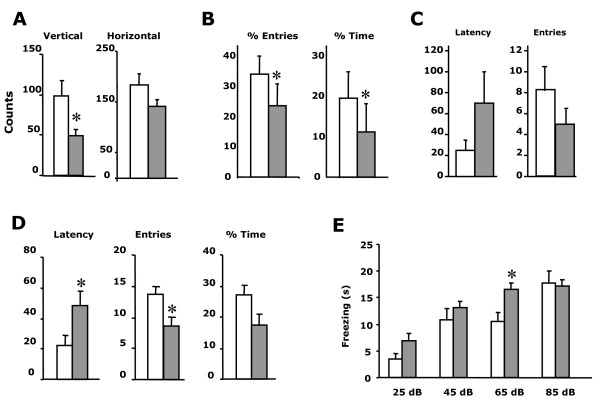
**Neurobehavioral phenotype**. **A**. *Locomotor activity*. A decrease in the vertical but not in horizontal locomotor activity measured in the actimetry box was observed in *Gtf2i*^*+/Δex*2 ^mice (*P *= 0.03). **B**. *Elevated Plus Maze*. Higher anxiety level was manifested in the *Gtf2i*^*+/Δex*2 ^indicated by the reduced percentage of entries in the open arms (*P *= 0.001) and in the time spent in open arms (*P *= 0.0089). **C**. ***Open Field ***. Histograms represent latency (in seconds) and the number of entries in central zone. **D.***Lit/dark box*. *Gtf2i*^*+/Δex*2 ^mice showed a higher anxiety level revealed by increased latency of the first entry (*P *= 0.01), the lower activity (*P*= 0.02) and the time spent into the lit compartment. **E**. **Acoustic sensitivity**. Enhanced sensitivity to an acute tone of 2800 Hz emitted at 65dB was observed in *Gtf2i*^*+/Δex*2 ^mice demonstrated by a longer lasting freezing behavior (*P *= 0.01). Freezer response was similar between genotypes at 85, 105 and 125 dB confirming a plateau of response. *Gtf2i*^*+/+*^, open; *Gtf2i*^*+/Δex*2^, grey squares. Each genotype groups are composed by males (n = 15). All values are expressed as mean ± s.d.

To evaluate a possible involvement of TFII-I in the psychomotor and neurobehavioral WBS phenotype, *Gtf2i*^*+/+*^, *Gtf2i*^*+/Δex*2 ^mice (n = 15 males per group) were evaluated in several paradigms. Motor coordination (wire hanging), locomotor activity (actimetry boxes and open-field) and anxiety-related behaviors (lit-dark box; elevated plus maze) were first explored. A significant decrease in the vertical but not in horizontal locomotor activity (Figure 4A) was evident in *Gtf2i*^*+/Δex*2 ^mice related to a decreased exploratory activity despite normal motor coordination (see Additional file 5).

Increased levels of anxiety were remarked in *Gtf2i*^*+/Δex*2 ^mice in the different paradigms used. Statistical differences were observed in the elevated plus maze (decreased percentage of entries and time in the open arms) (Figure 4B). In the open fild paradigm, no significant differences could be reported probably due to a major dispersion of results but we could observe a completed freezing of heterozygous animals in the central zone (initial site to the experiment) without any exploratory movement and later on a minor number of entries in the central zone suggesting all together a increase level of anxiety (Figure 4C). In the lit/dark box significant differences were found in the increased latency of the first entry and in the decreased number of entries, with the same tendency but not significant shorter permanence in the lit compartment (Figure 4D).

Sound intolerance was also measured by evaluating the time of freezing behavior at different sound intensities. *Gtf2i*^*+/Δex*2 ^mice showed a significantly lower threshold for sound intolerance, suggestive of the presence of algiacusis and/or hyperacusis already at 65 dB (Figure 4E).

No significant differences among genotypes were found in thermal nociceptive thresholds (tail immersion and hot plate tests), active avoidance and social behavior (intruder test) (see Additional file 5).

In summary, a significant phenotype was obvious in *Gtf2i*^*+/Δex*2 ^animals with decreased exploratory activity, higher anxiety and a lower threshold for sound intolerance.

## Discussion

In an attempt to create mouse models for WBS, we have generated a mutant mouse with an in frame deletion of exon 2 of *Gtf2i *resulting in the expression of a short TFII-I protein lacking the initial 140 amino acids that could function in some pathways as a lost of function allele. A remarkable neurobehavioral phenotype was evident in heterozygous mutant animals consisting in decreased exploratory activity despite normal motor coordination, enhancer anxiety and a low threshold for sound intolerance. Homozygous mutants showed a reduced viability early in development, with death before E8.5. However, the small proportion of surviving *Gtf2i*^Δex2/Δex2 ^mice, only 8% developed normally to adulthood with normal pre and postnatal growth. Our findings indicate that complete TFII-I activity is essential for cell proliferation during early embryogenesis and that such requirement can be only partially compensated in a small percentage of cases, possibly by other TFII-I family members. However, complete TFII-I activity seems not necessary for late fetal and postnatal development although its deficiency leads to specific neurological features.

Heterozygous *Gtf2i*^+/Δex2 ^MEFs proliferated well, but their growth rates were significantly slower and failed to growth dense cultures when compared to wild-type and homozygous *Gtf2i*^Δex2/Δex2 ^cells. Moreover, quiescent *Gtf2i*^+/Δex2 ^MEFs did not re-enter the cell cycle upon mitogenic stimuli as efficiently as their normal counterparts, suggesting a signaling defect mediated by the TFII-I-containing higher-order protein complexes. Therefore, we propose that the presence of the Δ140TFII-I protein leads to an abnormal composition of the TFII-I complexes with different consequences in the heterozygous or homozygous state. The leucine zipper motif is required for controlling the extent of complex formation and consequently its nuclear function [21]. Our results underscore the *in vivo *relevance of the N-terminal part of TFII-I in appropriate stoichiometric concentrations.

Based on clinical-molecular correlations in patients with partial deletions, *GTF2I *and *GTF2IRD1 *have been proposed as strong candidates for the craniofacial and some neurological features of WBS, while *CYLN2 *is thought to contribute to the neurological and cognitive phenotype [6-8]. Mouse models support these hypotheses. *Cyln2*^-/- ^and *Cyln2*^+/- ^mice present with mild structural brain abnormalities, hippocampal dysfunction, and deficits in motor coordination [23]. Three targeted *Gtf2ird1 *and one *Gtf2i *mice have been reported. The clear role of *Gtf2ird1 *in craniofacial abnormalities is clearly demonstrated in two of the models [10,12] while the other displayed reduced aggression and natural fear response, and increased social interaction combined with impaired amygdale-based learning [11]. However, haploinsufficiency for *CYLN2*, *GTF2IRD1 *and *ELN *alone or in combination cannot explain all the clinical features of WBS [23]. We show here that the deficiency of TFII-I by ablation of the first 140 amino acids in mice results in a characteristic phenotype related to the WBS features. *Gtf2i*^+/Δex2 ^and *Gtf2i*^Δex2/Δex2 ^mice display craniofacial abnormalities, with a shorter but symmetric snout and apparent midface hypoplasia. Interesting, haploinsufficiency of both *Gtf2i *and *Gtf2ird1 *does not result in additive or synergistic effects in craniofacial phenotype [13] as we could suppose due the fact that TFII-I and BEN share functional domains and expression patterns and are known to interact to each other. However, it is possible that a phenotype seen in the single-gene mutant could be modified in the deletion model due to complementary elects of the other genes in the deletion.

Most WBS individuals show odynacusis along with auditory allodynia; true hyperacusis, meaning a lowered hearing threshold, and auditory fascinations are also present although only in 5-10% of cases [24]. The origin of these highly penetrant auditory symptoms in WBS is not yet understood. The normal speech discrimination and the lack of abnormal loudness growth function in electrophysiological studies suggested abnormalities in central auditory processing [25]. Very recently, Li and collaborators demonstrate that mice with a half proximal deletion, including *Gtf2i *(PD) recapitulates some of the features of WBS patients like, social interaction phenotype or abnormal sound sensitivity [13]. Until this work, no behavioral phenotypes in *Gtf2i *mutant mice have been reported. Here we demonstrate that the encoded TFII-I molecule is a mean player in the abnormal sound sensitivity phenotype. *Gtf2i*^+/Δex2 ^animals showed clear odynacusis, manifested by a lowered pain threshold for loud sound (evident at 65dB), thus indicating that deletion of *GTF2I *is likely responsible for the similar auditory phenotype in WBS.

## Conclusions

Our observations in the *Gtf2i*^Δex2 ^mouse models illustrate that *GTF2I *associates with some of the main phenotypic features of WBS. A remarkable neurobehavioral phenotype consisting in decreased exploratory activity despite normal motor coordination, enhancer anxiety and a low threshold for sound intolerance was evident in heterozygous mutant animals. As shown by previous studies in human and mouse, no single gene appears to be responsible for all the craniofacial or neurocognitive features of WBS [3,6-8,10-12,23]. Instead, additive effects of haploinsufficiency at several genes appear to be required, and we propose that *GTF2I *is one of the main players. Our data strongly suggest that the N-terminus of TFII-I is essential for formation of higher-order protein complexes, thus playing an important role in the final functions of TFII-I in development and neurocognition.

## Competing interests

The authors declare that they have no competing interests.

## Authors' contributions

Conceived and designed the experiments, analyzed the results and wrote the manuscript: VC, LPJ. Performed the experiments: Molecular and cellular biology and histology: JL, SP, MS, MZ, VC. Targeting vectors: MCV, LPJ. Mouse generation, maintenance and genotyping: JL, SP, CC, IB, JLB. Behavioral phenotype: EA, JL, RM. Mouse pathology: PD. Stereological analysis of the hippocampus: AS, NS. All authors read and approved the final manuscript.

## Pre-publication history

The pre-publication history for this paper can be accessed here:

http://www.biomedcentral.com/1471-2350/11/61/prepub

## Supplementary Material

Additional file 1 **Methods**. A more detailed description of methodology employed to develop the project. Click here for file

Additional file 2 **Figure S5.-DNA binding and protein-protein interaction. A**. *ΔTFII-I binds DNA*. PCR amplification from chromatine immunoprecipitated with anti-TFII-I Ab. ChIP assays were performed into wild-type, heterozygous and homozygous MEFs. A specific band is amplified corresponding to *Birc1F *promoter. No amplification was observed in the IgG immunoprecipitates of in the negative control *Renl*. B. *TFII-I does not dimerize with ΔTFII-I*. Whole-cell lysates of cotransfected COS7 were subjected to IP with anti-V5 Ab. Immunoprecipitates were detected with anti GST Ab (upper panel). Cell lysates of transfected wild-type and heterozygous MEFs were subjected to GST pulldown assays. Levels of endogenous TFII-I forms were detected by immunobloting with anti-TFII-I Ab (lower panel). C. *PARP1A does not immunoprecipitate with ΔTFII-I*. Whole-cell lysates of cotransfected COS7 were subjected to IP with anti-V5 Ab. Immunoprecipitates were detected with anti GST Ab Click here for file

Additional file 3 **Figure S6 -mRNA expression in *in vitro*assays. A**. *Relative levels of mRNAs *Histogram representing the relative levels of mRNA expression of different genes (white, *Gapdh*; grey, *Gtf2i*; striped, *Cyclin D; *punctated, c-*fos*; black, luciferase under control of c-*fos *promoter) after transient transfection of COS7 with plasmids expressing the indicated protein. mRNA levels have been normalized respect to expression levels presented by a mock transfected COS7 cells. Note the increase expression of *Gtf2i *after transfection. Only in positive control (cells transfected with plasmid expressing human GTF2I-Δ isoform) and in cells transfected with murine GTF2I-Δ isoform we could observe 4-fold increased expression for *Cyclin D1*. All the assays have been made by triplicate. B. *Endogenous mRNA levels*. Relative endogenous mRNA levels of indicated genes in *Gtf2i*^+/+ ^arrested (black), *Gtf2i*^*Δex*2/*Δex*2 ^arrested (striped), *Gtf2i*^+/+ ^stimulated (grey) and *Gtf2i*^*Δex*2/*Δex*2 ^stimulated (white) MEFs. We could not observe any difference between the conditions analyzed. All the assays have been made by triplicate. Click here for file

Additional file 4 Table S2: Homozygous mice Fertility Click here for file

Additional file 5 **Figure S7-Neurobehavioral phenotype.** A. *Motor coordination*. Any difference could be observed between wild-types and heterozygous mutant mice in all the parameters tested. B. *Hot plate*. The histogram represents the tail flick measured in seconds. C. *Tail immersion*. The histogram shows the time in seconds until the jumping D. *Active avoidance*. No differences could be appreciated in the active avoidance test among genotypes. E. *Social interaction*. Resident-intruder tests. Any of these behavioral analysis reveled significant differences between genotypes. *Gtf2i*^+/+ ^open square; *Gtf2i*^*+/Δex2*^, grey square. Each genotype groups are composed only by males (n = 15). Click here for file
