# Supplementary material for: Essential role of the N-terminal region of TFII-I in viability and behavior
Source: BMC Med Genet. 2010 Apr 19;11:61. doi: 10.1186/1471-2350-11-61 (PMC2865459; doi:10.1186/1471-2350-11-61)
Supplement: Additional file 1 — Methods. A more detailed description of methodology employed to develop the project. [file 1471-2350-11-61-S1.DOC]

**Additional File 1: Methods**

**Gene targeting and genotyping strategies**

Mouse *Gtf2i* cDNAs and genomic sequences were cloned by plate hybridization from a mouse testis cDNA library and a lambda genomic library, respectively. For excision of the second exon of *Gtf2i*, which contains the initiation start codon, we subcloned upstream and downstream genomic fragments in a plasmid containing the *PGK-neo* cassette (Figure 1A). The resulting targeting vector, p856, was linearized and electroporated into mouse R1 ES cells, and recombinant clones were selected in the presence of G418. Seven G418^R^ clones were identified by Southern hybridization using probes external to the targeting vector and PCR strategies. Recombinant clones were used to generate chimeric mice by aggregation to CD1 morulae. *Gtf2i^+/Δex2^* mice were intercrossed to generate mouse colonies in the 129Sv × CD1 background or backcrossed to generate a pure C57BL/6J colony. The experiments described in this manuscript were carried out with animals in the 129Sv × CD1 background.

Genotyping of *Gtf2i* mutant mice was carried out by PCR amplification. To identify wild-type allele (377 bp) we used primers m*Gtf2i*-I1F (forward) and m*Gtf2i*-I1R (reverse) (See below). To identify the mutant (*Δex2)* allele (309 bp) we added a second reverse primer m*Gtf2i*-neo to the amplification reaction. Primer sequences are included below. The annealing temperature was 60ºC and they were amplified for 30 cycles.

**mRNA expression analysis**

Total RNA from cells and tissue samples of mice was isolated using the TRIZOL reagent (Invitrogen) for RT-PCR analysis and mRNA quantification, cDNA was prepared from 1 µg total RNA using random hexamers and SuperScript II RNase H- reverse transcriptase (Invitrogen). The cDNA solution was subjected to 25-30 cycles of PCR amplification by using specific primers (see below). Absence of genomic DNA contamination was determined by the non-amplification of a small genomic fragment using genomic DNA as positive control. RT–PCR products were subject to electrophoresis in 2% agarose gel, stained with ethidium bromide and visualized. Relative quantification was obtained using the software Quantity One (Version 4.3.1 Biorad). Normalization of expression were performed with *Actin* and/or *Hsp70* genes.

**Cell Culture and Immunocytofluorescence**

We obtained primary MEFs from E13.5 embryos as previously described and cultured them in Dulbecco's modified Eagle's medium (DMEM, Life Technologies) supplemented with 2 mM glutamine, 1% penicillin/streptomycin and 10% calf bovine serum (CBS). For proliferation assays, we plated 5 X10^4^ cells on six-well plates. For colony formation assays, we seeded 4 X10^3^ cells on 10-cm plates and cultured them for 2 weeks. We stained the plates with methylene blue and scored the colonies. To analyze S-phase entry, we incubated 10^6^ cells per 10-cm dish in DMEM supplemented with 0.1% FBS for 60h. We added DMEM supplemented with 10% CBS and then collected cells at the indicated times, stained them with propidium iodide and analyzed by fluorescence-activated cell sorting using a Coulter flow cytometer.

To detect endogenous TFII-I immunocytofluorescence was performed in primary MEFs. The cells were cultured on sterile glass cover slips mounted in 3.5 cm culture dishes. Cells were fixed with 4% paraformaldehyde in PBS 20 min at room temperature and permeabilized with 0.1% Triton-X-100 in PBS for 5 min at room temperature. Nonspecific binding sites were blocked with 3% bovine serum albumin solution in PBS and cells were stained with the anti-TFII-I rabbit polyclonal antibodies in 1:3000 dilution. The secondary sheep anti-rabbit FITC conjugated IgGs (Sigma, C2306) were used in 1/200 dilution. Nuclear DNA was additionally stained with Hoechst 33258 (Molecular Probes) at a concentration 0.5 μg/ml for 10 min at room temperature and then washed three times with PBS. Cover slips were mounted on microscopic glass slides using Vectashield (Vector Laboratories) to prevent fading of the fluorescent dye. Microphotographs were taken using an Olympus BXS1 microscope with an epifluorescence and phase-contrast optics equipped with the Olympus DP71 camera and using the software Cell^B^ Digital Imaging system.

**ChIP , IPs and GST pulldowns**

ChIP analysis was performed in wild-type, heterozygous and homozygous MEFs following standard procedures. Anti-TFII-I rabbit polyclonal antibody was used at 1:150 dilutions. Amplification of sequences for *Reln* promoter has been used as negative control. Promoter sequence for *Birc1f* has been used as positive target. Primer sequences are included below.

For protein-protein interactions COS7 cells were co-transfected using Lipofectamine 2000 (Sigma) following manufactur's recommendations with plasmid expressing WT-TFII-I-GST or PARP-GST together with WT-TFII-V5 (as positive control) or ΔTFII-I-V5. For immunoprecipitation we used anti-V5 antibody (Ambion) and western blot were develop with anti-GST antibody (Sigma). To test dimerization wild-type and heterozygous MEFs were transiently transfected with plasmid expressing WT-TFII-I-GST fusion protein. After immunoprecipitation using glutation beads and following standard protocol, western blot were reveled with anti-TFII-I rabbit polyclonal antibodies in 1:8000 dilution. Secondary anti-rabbit IRDye800 or Cy5.5 conjugated antibodies were used following Odyssey^TM^ Westen Blotting Protocols.

**Primer Sequences**

**Name Sequence**

**Genotyping and Southern blot prove**

m*Gtf2i*-I1F 5’-GGTTGGGTTCTCTGACATTTCTAT-3’

m*Gtf2i*-I1R 5’-CACGCATGCTGTAATACTTGG-3’

m*Gtf2i*-neo 5’-CAAGCACTGGCTATGCATGTATA-3’

m*Gtf2i*-I1PF 5’-CATCCCACTTGGCTGTCAC-3’

m*Gtf2i*-I1PR 5’-ATACTCCCCATTCCCACACA-3’

***Gtf2i* RNA expression, analysis of isoforms and cloning of cDNAs**

m*Gtf2i*-1F 5’-CCTACACTCGCTTGCTGTCC-3’

m*Gtf2i*-2F 5’-AAGTAGCGATGTCTGCCTTG-3’

m*Gtf2i*-4R 5’-TTCATCTTATGCATCTCGGCGG-3’

TFIIWT 5’-CACCATGGGATCTGATAAA-3’;

TFIIN140 5’-CACCATGCTGCGCGACCAGTCG-3’;

TFIIR 5’ TGCAGTCGTCGAGTGCTCCGACTT-3’

**RNA expression of *Gtf2i* targets and controls**

*CyclinD1*F 5’-AGTGCAAGGCCTGAACCTGAGGAG-3’

*CyclinD1*R 5’-TCAGATGTCCACGTCCCGCACGTC-3’

*B-Actin*F 5’-TCCTTCCTGGGCATGGAGTCC-3’

*B-Actin*R 5’-GAGGAGCAATGATCTTGATCTTC-3’

*C-fos*F 5’-CATGGGCTCTCCTGTCAACAC-3’

*C-fos*R 5’-TCTCCGTTTCTCTTCCTCTTC-3’

*Gapdh*-F 5’- TGGTATCGTGGAAGGACTCATGAC-3’

*Gapdh*-R 5’- ATGCCAGTGAGCTTCCCGTTCAGC-3’

*Hsp70*-F 5’- ACAGTGGTGCCTACCAAGAAG-3’

*Hsp70*-R 5’- CTTGTCTTCAGCTGTCACTCG-3’

*mGtf2i*-15F 5’- TTTTCCAGTCCCATGTTGAA-3’

*mGtf2i*-24R 5’- CCACTTTCTGCTTCAGGTCT-3’

**ChIP**

*Reln-*F 5’- ACCACTCCAGGTGGCAATC-3’

*Reln-*R 5’- CGAGCCCTTTCCAGTCAG-3’

*Birc1f-*F 5’- GGGCTAAGTGTGGCATCCT-3’

*Birc1f-*R 5’- GGCAAGCTTTCTCTGTGTGG-3’

**Skull size**

To calculate the skull size many parameters has been quantified. We take 6 measures in a superior view, 3 for a longitudinal and 3 for the transversal planes. We also take 4 measures in a lateral view in order to calculate the skull volume. All measures are relative values.

**Stereological procedures**

Brains were removed and placed in fixative and after 4 weeks in fixative, brains were split into two hemispheres by a midsagittal section and processed for stereology, according to the procedure described previously [1]. Briefly, they were included in glycomethacrylate (Tecnovit 7100; Heraeus Kulzer, Werheim, Germany) and every other microtome-cut section (30 µm) was then collected on a gelatinized slide, stained with Giemsa, and mounted with Entellan New (Merck, Darmstadt, Germany). The shrinkage factor was calculated according to Madeira et al. [2].

**Region and Layer Boundaries**

Hippocampal formation was analyzed according to its main anatomical divisions: dentate gyrus (including hilus, granule cell layer, and molecular layer), CA3, and CA1 (strata oriens, pyramidale, and radiatum). The above mentioned regions were outlined according to the atlas of Paxinos (2001), based on noticeable cytoarchitectural differences.

**Stereological Procedure**

Volume and neuronal number estimations were performed using StereoInvestigator software (MicroBrightField, Williston, VT) and a camera (DXC390; Sony, Tokyo, Japan) attached to a monitorized microscope (Axioplans, Zeiss, Oberkochen, Germany). Cavalieri’s principle was used to assess the volume of each region.

Briefly, every 8^th^ section was used and its cross-sectional area was estimated. For this, we randomly superimposed onto each area a test point grid in which the interpoint distance, at tissue level was 100µm for DG, 120 µm for CA1 and 150 µm for CA3. The volume of the region of interest was calculated from the number of points that fell within its boundaries and the distance between the systematically sampled sections. Average cell numbers were estimated using the optical fractionator method, as described elsewhere [3] and using a 100x immersion oil objective. Only the neurons that fell within the box where counted (without touching the red line). Cell packing density was defined as the number of neurons per unit of volume (µm^3^).

Briefly, the following sampling scheme was used:

1. Every 8^th^ section was measured;
2. Beginning at a random starting position, a grid of virtual three-dimensional boxes (20x20x20µm) that were equally spaced (the same grid as that for volume estimations) was superimposed within the predefined borders;
3. Neurons were counted whenever their nucleus came into the focus within the counting box. Neurons were differentiated from other cells on the basis of nuclear size (larger in neurons, and the shape of their perikarya attributable to dendritic emergence [4].

Coefficients of error were computed by the software according to the formulas of Gundersen et al. (1999) for cell numbers and Gundersen and Jensen (1987) for volume estimations [5, 6].

**Behavioral characterization of mice**

**Motor coordination.**

Motor coordination and neuromuscular strength were assessed by the wire hanging test. The apparatus consisted of a coat hanger (50 cm; 2-mm diameter) suspended from a stainless-steel bar resting on two vertical supports and elevated 32 cm above a sawdust covered surface. Mice were suspended by its forepaws during 5 sec and neuromuscular strength was scored according to the following system: 0, fell off; 1, hung onto the bar with two forepaws and one hind paw; 2, hung onto the bar with the four paws; 3, escaped to one of the supports. The test was performed twice for each mouse. Immediately after neuromuscular strength evaluation, motor coordination was assessed. Latency to fall, to arrive to the terminal part of the hanging was measured from the time animals were placed hanging by its forepaws on the wire until it fell for a maximum time of 1 min. Number of travels in the hanging was also counted.

**Locomotor activity**

Locomotor activity responses were evaluated during 30 min for 3 consecutive days by using locomotor activity boxes (9 x 20 x 11 cm) (Imetronic, Bordeaux, France). The boxes were provided with two lines of photocells, one 2 cm above the floor to measure horizontal activity, and the other located 6 cm above the floor to measure vertical activity (rears), in a low luminosity environment (5 lux).

**Lit-dark box**

Mice were individually exposed for 5 min to a box consisting of a small compartment (15 cm x 20 cm x 25 cm) with black walls and a black floor dimly lit (5 Lux), connected by a 4-cm-long tunnel to a large compartment (30 cm x 20 cm x 25 cm) with white walls and white floor, intensely lit (500 Lux). Lines were drawn on the floor of both compartments to allow measurement of locomotor activity by counting the number of squares (5 cm x 5 cm) crossed. Floor lines separated the lit compartment into three equal zones, from the tunnel to the opposite wall, designated as proximal, median and distal zone. Each animal was placed in the dark compartment facing the tunnel at the beginning of each session. The time spent in each compartment, the locomotor activity and the number of visits into each zone of the lit compartment was all recorded.

**Elevated plus-maze**

The elevated plus-maze consisted of a black apparatus with four arms (16 cm long x 5 cm wide) set in cross from a neutral central square (5 cm x 5 cm). Two opposite arms were delimited by vertical walls (closed arms), whereas the two other opposite arms had unprotected edges (open arms). The maze was elevated 30 cm above the ground and placed in indirect light (100 Lux). At the beginning of the 5-min observation session, each mouse was placed in the central neutral zone, facing one of the open arms. The total numbers of visits to the closed arms and the open arms, and the cumulative time spent in the open arms and the closed arms were then observed on a monitor through a video camera system (ViewPoint). An arm visit was recorded when the mouse moved both forepaws into the arm.

**Open field**

Each animal was placed in an open-field apparatus consisting of a rectangular area (70 cm wide x 90 cm long x 60 cm high) under bright illumination (500 Lux). Black lines were drawn on the white floor of the apparatus to allow measurement of locomotor activity by counting the number of squares (10 cm x 10 cm) crossed. The parameters measured during the observation session of five min were the latency of crossing the first two squares from the central square where the mouse is initially placed; the number of squares crossed in the peripheral and central area of the field; entries into the central area; rearings; grooming bouts; defecation and urination events. Mice were exposed to the test for three consecutive days.

**Nociceptive thresholds**

Thermal nociceptive thresholds were evaluated by the tail immersion and the hot plate test and mechanical nociception was measured with the tail pressure test. For the tail immersion test, mice were maintained in a cylinder and their tail immersed in water at 50 °C; latency to tail withdrawal was measured. In the hot plate test, mice were placed on a hot surface heated at 52 °C and the latency for paw licking and jumping was recorded. For the tail pressure, increasing pressure was applied to the tail of mice (tip diameter 1 mm) until a withdrawal response was elicited and the pressure exerted was recorded.

**Active avoidance procedure**

Mice were trained to avoid an aversive stimulus associated with the presentation of a conditioned stimulus (CS) in a two-way shuttle box apparatus (Panlab, Barcelona, Spain). The apparatus consists of a box with two compartments (20×10 cm) connected by a 3×3-cm door. A light (10 W) switched on in the compartment in which the mouse was placed was used as a CS. The CS preceded by 5 sec the onset of the unconditioned stimulus (US) and overlapped it for 25 sec. Using this procedure, the light was presented in the compartment for 30 sec (5 sec alone and 25 sec together with the US). At the end of the 30-sec period, both CS and US were automatically turned off. The US was an electric shock (0.2 mA) continuously applied to the grid of the floor. A conditioned response was recorded when the animal avoided the US by changing from the compartment where the animal received the CS into the opposite compartment within the 5 sec after the onset of the CS. If animals failed to avoid the shock, they could escape it by crossing during the US (25 sec) and it was recorded as unconditioned response. Between each trial session, there was an inter-trial interval of 30 sec. The ratio of conditioned responses respect to the total number of changes of compartment during the whole session was determined. Animals were subjected to five daily 100-trial active avoidance sessions. Each day the mice were placed in the shuttle box 10 min before the start of each session to allow them to explore the box and to habituate to the apparatus.

**Resident–intruder procedure**

This social interaction test evaluates aggressive behavior in rodents. Resident mice were housed individually for 10 weeks prior to the beginning of the experimental procedure. Intruder animals of a similar age and weight were housed five per cage. Each session consisted of putting together resident and intruder mice for a period of 4 min. Then, the attack frequency and duration based on the presence of an attack or menace (beating tail) by the resident were measured for each 10-sec period. Animals received two training sessions in the morning and two test sessions in the afternoon.

**Acoustic sensitivity**

The apparatus consists of a dark box with a transparent wall which allows the observation of the animals that is connected to a sound generator. Mice were individually placed in the cage during 1 min in order to habituate them to the new environment. Then, animals were exposed to a 2800 Hz tone at 25 dB during 30 sec. The freezing behavior and physical signs such as ptosis or piloerection were recorded. After a recovery period of 60 min, animals were exposed to three further sessions at 45, 65 and 85 dB, respectively, following the same time schedule.

**Statistical Analysis**

Results are expressed as group means ± SD. Data analysis were performed with SPSS, with alpha at *p*< 0.05. Analysis of variance (ANOVA) with Bonferroni and Tukey *post-hoc* tests was used. Normality of data was tested (Shapiro-Wilk normality test).

**References**

1. Keuker JI V-HGaFE: **How to use the optical fractionator:an example based on the estimation of neurons in the hippocampal CA1 and CA3 regions of tree shrews**. *Brain Res Protoc* 2001, **7**:211-221.

2. Madeira MD, Pereira A, Cadete-Leite A, Paula-Barbosa MM: **Estimates of volumes and pyramidal cell numbers in the prelimbic subarea of the prefrontal cortex in experimental hypothyroid rats**. *J Anat* 1990, **171**:41-56.

3. West MJ, Slomianka L, Gundersen HJ: **Unbiased stereological estimation of the total number of neurons in thesubdivisions of the rat hippocampus using the optical fractionator**. *Anat Rec* 1991, **231**(4):482-497.

4. Peinado MA, Quesada A, Pedrosa JA, Martinez M, Esteban FJ, Del Moral ML, Peinado JM: **Light microscopic quantification of morphological changes during aging in neurons and glia of the rat parietal cortex**. *Anat Rec* 1997, **247**(3):420-425.

5. Gundersen HJ, Jensen EB: **The efficiency of systematic sampling in stereology and its prediction**. *J Microsc* 1987, **147**(Pt 3):229-263.

6. Gundersen HJ, Jensen EB, Kieu K, Nielsen J: **The efficiency of systematic sampling in stereology--reconsidered**. *J Microsc* 1999, **193**(Pt 3):199-211.
