# Supplementary material for: Essential role of the N-terminal region of TFII-I in viability and behavior
Source: BMC Med Genet. 2010 Apr 19;11:61. doi: 10.1186/1471-2350-11-61 (PMC2865459; doi:10.1186/1471-2350-11-61)
Supplement: Additional file 2 — Figure S5.-DNA binding and protein-protein interaction. A. ΔTFII-I binds DNA. PCR amplification from chromatine immunoprecipitated with anti-TFII-I Ab. ChIP assays were performed into wild-type, heterozygous and homozygous MEFs. A specific band is amplified corresponding to Birc1F promoter. No amplification was observed in the IgG immunoprecipitates of in the negative control Renl. B. TFII-I does not dimerize with ΔTFII-I. Whole-cell lysates of cotransfected COS7 were subjected to IP with anti-V5 Ab. Immunoprecipitates were detected with anti GST Ab (upper panel). Cell lysates of transfected wild-type and heterozygous MEFs were subjected to GST pulldown assays. Levels of endogenous TFII-I forms were detected by immunobloting with anti-TFII-I Ab (lower panel). C. PARP1A does not immunoprecipitate with ΔTFII-I. Whole-cell lysates of cotransfected COS7 were subjected to IP with anti-V5 Ab. Immunoprecipitates were detected with anti GST Ab [file 1471-2350-11-61-S2.PPT]

## Slide 1
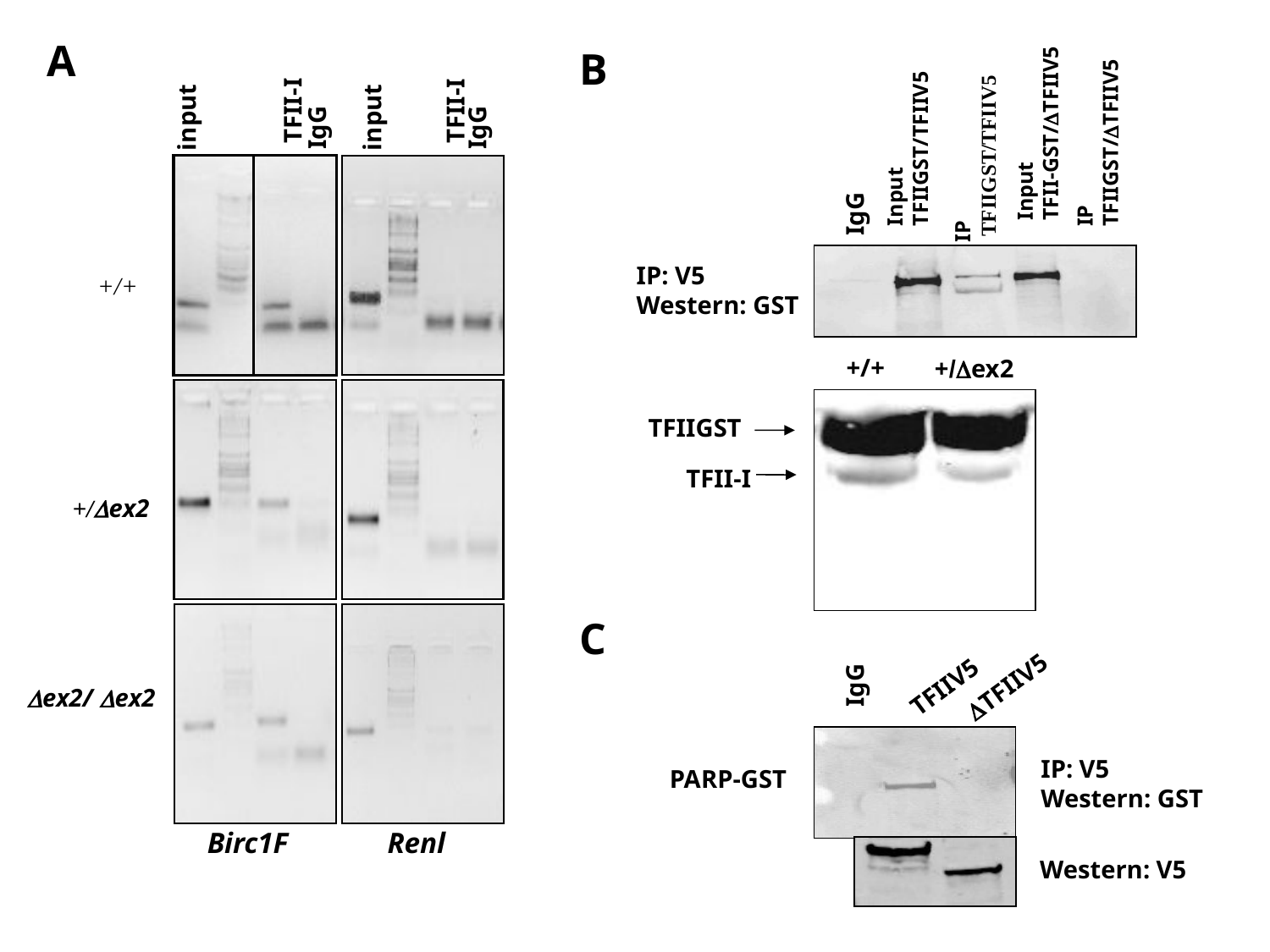

A
B
TFII-I
TFII-I
input
input
Input
TFII-GST/ΔTFIIV5
IgG
IgG
IP
TFIIGST/ΔTFIIV5
Input
TFIIGST/TFIIV5
IP
 TFIIGST/TFIIV5
IgG
IP: V5
Western: GST
+/+
+/+
+/Δex2
TFIIGST
TFII-I
+/Δex2
C
IgG
ΔTFIIV5
TFIIV5
IP: V5
Western: GST
PARP-GST
Western: V5
Δex2/ Δex2
Birc1F
Renl
