# Supplementary figures and images for: Essential role of the N-terminal region of TFII-I in viability and behavior
Source: BMC Med Genet. 2010 Apr 19;11:61. doi: 10.1186/1471-2350-11-61 (PMC2865459; doi:10.1186/1471-2350-11-61)

A

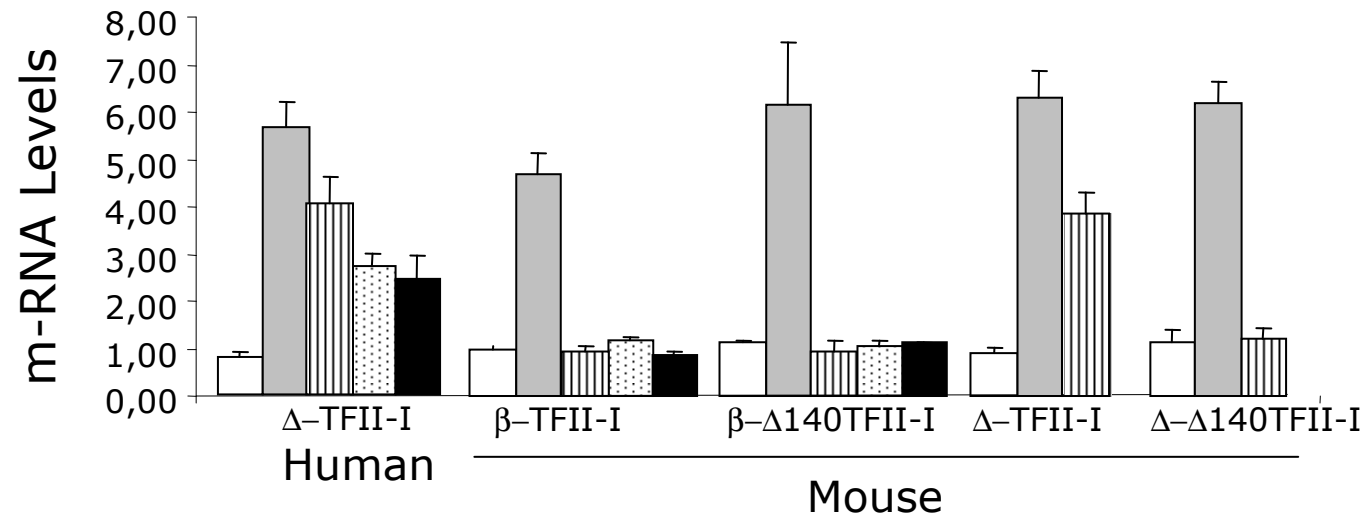

B

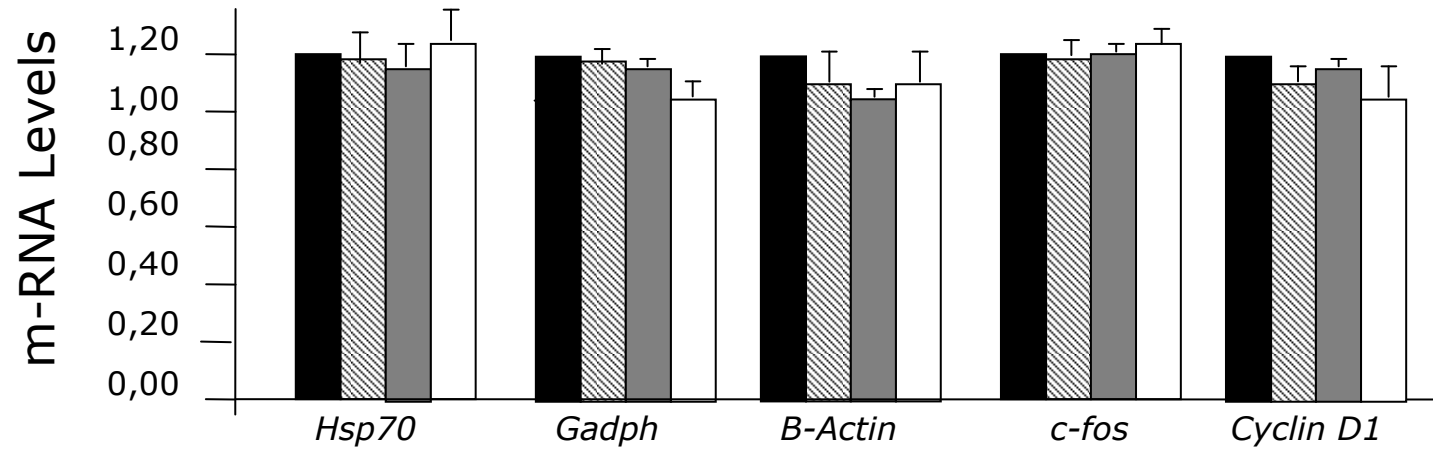

Supplement: Additional file 3 — Figure S6 -mRNA expression in in vitroassays. A. Relative levels of mRNAs Histogram representing the relative levels of mRNA expression of different genes (white, Gapdh; grey, Gtf2i; striped, Cyclin D; punctated, c-fos; black, luciferase under control of c-fos promoter) after transient transfection of COS7 with plasmids expressing the indicated protein. mRNA levels have been normalized respect to expression levels presented by a mock transfected COS7 cells. Note the increase expression of Gtf2i after transfection. Only in positive control (cells transfected with plasmid expressing human GTF2I-Δ isoform) and in cells transfected with murine GTF2I-Δ isoform we could observe 4-fold increased expression for Cyclin D1. All the assays have been made by triplicate. B. Endogenous mRNA levels. Relative endogenous mRNA levels of indicated genes in Gtf2i+/+ arrested (black), Gtf2iΔex2/Δex2 arrested (striped), Gtf2i+/+ stimulated (grey) and Gtf2iΔex2/Δex2 stimulated (white) MEFs. We could not observe any difference between the conditions analyzed. All the assays have been made by triplicate. [file 1471-2350-11-61-S3.PDF]

**A**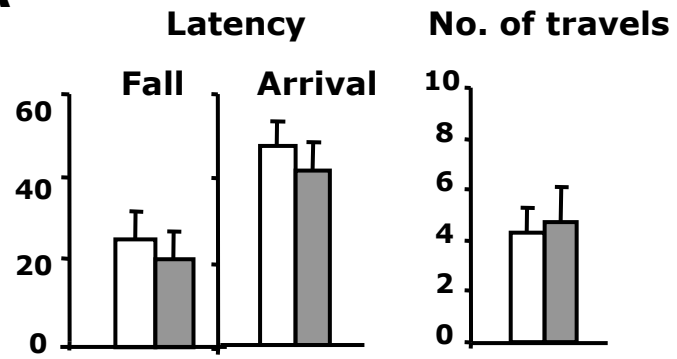**B**

Tail flick (s)

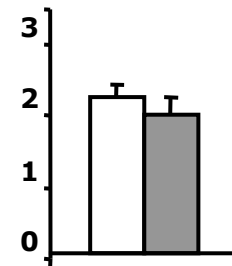**C**

Jumping (s)

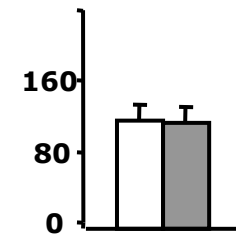**D**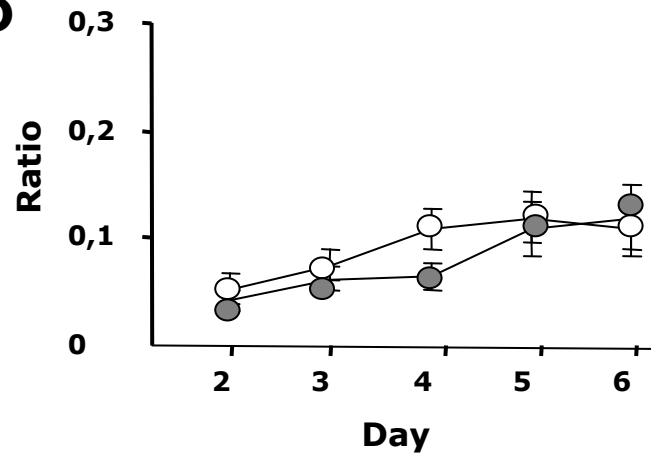**E**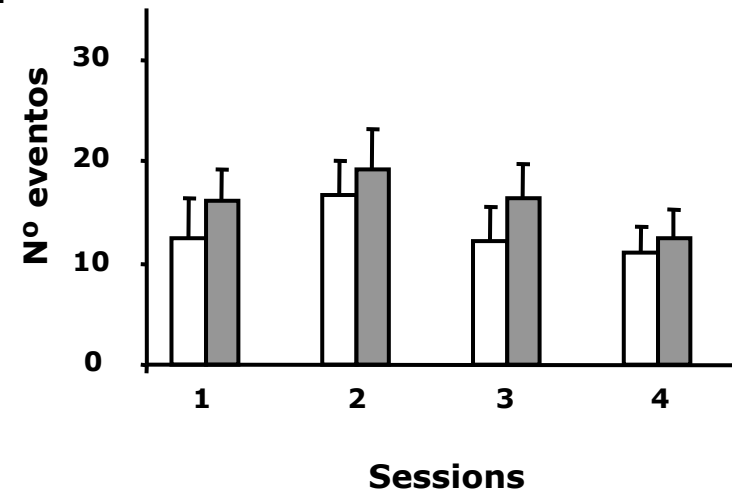

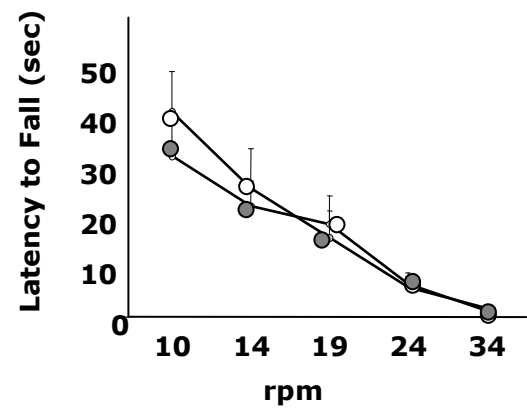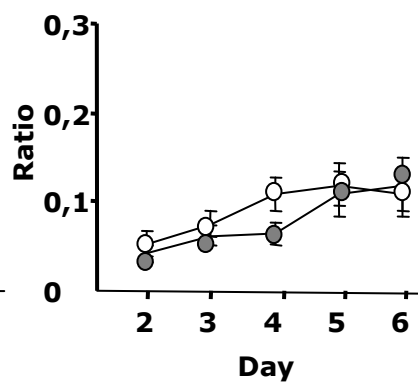

Supplement: Additional file 5 — Figure S7-Neurobehavioral phenotype. A. Motor coordination. Any difference could be observed between wild-types and heterozygous mutant mice in all the parameters tested. B. Hot plate. The histogram represents the tail flick measured in seconds. C. Tail immersion. The histogram shows the time in seconds until the jumping D. Active avoidance. No differences could be appreciated in the active avoidance test among genotypes. E. Social interaction. Resident-intruder tests. Any of these behavioral analysis reveled significant differences between genotypes. Gtf2i+/+ open square; Gtf2i+/Δex2, grey square. Each genotype groups are composed only by males (n = 15). [file 1471-2350-11-61-S5.PDF]
