# Supplementary material for: Essential role of the N-terminal region of TFII-I in viability and behavior
Source: BMC Med Genet. 2010 Apr 19;11:61. doi: 10.1186/1471-2350-11-61 (PMC2865459; doi:10.1186/1471-2350-11-61)
Supplement: Additional file 4 — Table S2: Homozygous mice Fertility [file 1471-2350-11-61-S4.DOC]

**Additional File 4**

**Table 2. Homozygous mice Fertility.**

| **Mating** | | **Mean Nº litters** | **Mean Nº pups per litter** | | | |
| --- | --- | --- | --- | --- | --- | --- |
| Male | Female |  | 1 | 2 | 3 | 4 |
| *Δex2/Δex2* | +/+ | 3.2±0.83 | 7.8±1.6 | 6.0±1.87 | 3.5±1.29 | 2.5±0.7 |
| *Δex2/Δex2* | *+/Δex2* | 3±1 | 5.6±1.14 | 2.5±1.29 | 2.0±1.0 | 1.5±0.7 |
| +/+ | *Δex2/Δex2* | 2.4±1.14 | 6.8±1.9 | 4.75±0.95 | 7.5±0.7 | 1 |
| *+/Δex2* | *Δex2/Δex2* | 2±0.7 | 3.6±0.89 | 3.0±0.81 | 1.5±0.7 | 1 |
